# High primary resistance to metronidazole and levofloxacin, and a moderate resistance to clarithromycin in *Helicobacter pylori* isolated from Karnataka patients

**DOI:** 10.1186/s13099-019-0305-x

**Published:** 2019-05-13

**Authors:** Vignesh Shetty, Binit Lamichhane, Chin Yen Tay, Ganesh C. Pai, Ramachandra Lingadakai, Girisha Balaraju, Shiran Shetty, Mamatha Ballal, Eng Guan Chua

**Affiliations:** 1 Enteric Diseases Division, Central Research Lab, Kasturba Medical College, Manipal Academy of Higher Education, Manipal, India; 2 Marshall Centre for Infectious Diseases Research and Training, School of Biomedical Sciences, University of Western Australia, Crawley, WA Australia; 3 Shenzhen Dapeng New District Kuichong People Hospital, Shenzhen City, Guangdong Province China; 4 Department of Gastroenterology & Hepatology, Kasturba Medical College, Manipal Academy of Higher Education, Manipal, Karnataka India; 5 Department of Surgery, Kasturba Medical College, Manipal Academy of Higher Education, Manipal, Karnataka India

**Keywords:** *Helicobacter pylori*, Antibiotic susceptibility, India, Agar dilution, Minimum inhibitory concentration

## Abstract

**Background:**

Due to increased prevalence of *H. pylori* antimicrobial resistance worldwide and more importantly the resistance patterns vary between different geographical regions, it is important to survey local *H. pylori* antibiotic resistance profile to provide physicians with more informed drug choices to better treat *H. pylori* infection. To our knowledge, this is the first study to examine the prevalence of antimicrobial resistance of *H. pylori* in Karnataka state of South India.

**Results:**

A total of 113 *H. pylori* strains were isolated from gastric biopsies and tested: 81.4% were resistant to metronidazole, 54.9% were resistant to levofloxacin, 20.4% were resistant to clarithromycin, 5.3% were resistant to tetracycline and 7.1% were resistant to amoxicillin. Multidrug resistance was detected in 59.3% of total isolated strains, among which 86.6% were resistant to at least both metronidazole and levofloxacin. In this study, 38 out of 113 *H. pylori* strains had been whole-genome sequenced. Based on the draft genomes, RdxA and/or FrxA inactivation mutations were found to present in 75% of metronidazole-resistant strains. Clarithromycin-resistant strains had mainly A2143G and G2224A mutations in the *23 rRNA* gene. While 87.1% levofloxacin-resistant strains had amino acid substitution mutations occurring predominantly at N87 and D91 in GyrA, novel mutations in the same protein including an insertion of five amino acid residues (QDNSV), immediately after the start codon, and a substitution mutation at R295 were identified.

**Conclusion:**

High primary resistance to metronidazole and levofloxacin, and a modest occurrence of clarithromycin resistance were revealed in *H. pylori* strains isolated from Karnataka patients. Therefore metronidazole-, levofloxacin- and clarithromycin-based triple therapies are not suitable as first-line treatment in Karnataka. Both amoxicillin and tetracycline can still be used to eradicate *H. pylori* infection in this region. We also revealed novel mutations in GyrA protein that possibly contribute to *H. pylori* resistance in levofloxacin, which merit further investigations.

**Electronic supplementary material:**

The online version of this article (10.1186/s13099-019-0305-x) contains supplementary material, which is available to authorized users.

## Introduction

*Helicobacter pylori* is a gastric pathogen that colonises human stomach, leading to clinical manifestations of chronic gastritis, peptic ulcer disease, gastric adenocarcinoma and mucosa associated lymphoid tissue (MALT) lymphoma [1]. Current standard *H. pylori* eradication regime, also known as triple therapy, comprises two antibiotics (amoxicillin and clarithromycin or metronidazole) and a proton pump inhibitor (PPI) [5]. However, due to surging prevalence of clarithromycin and metronidazole resistance in *H. pylori*, the treatment success rate of either clarithromycin-based or metronidazole-based triple therapy is merely 80% or less in certain global regions [2–6]. To improve *H. pylori* eradication rate, different treatment strategies have been implemented including concomitant, sequential and bismuth-based quadruple regimens, as well as the culture-based, susceptibility-guided treatment approach [7, 8].

While different antibiotics have been used for the treatment of *H. pylori* infection, the bacterium has developed several mechanisms to protect itself against the antimicrobial activities of levofloxacin, tetracycline and rifabutin, in addition to metronidazole and clarithromycin [9]. Metronidazole resistance is complex but is primarily due to the inactivation of RdxA, an oxygen-insensitive NADPH nitroreductase [10]. Additionally, it has been demonstrated that the level of metronidazole resistance attributed to RdxA inactivation can be enhanced further by FrxA nitroreductase [11]. For clarithromycin resistance, substitution mutations involving the adenine residues at positions 2142 and 2143 were frequently detected in the *23 rRNA* gene [12]. Furthermore, amino acid substitutions at positions 87 and 91 in DNA gyrase subunit A (GyrA) resulted in *H. pylori* resistance against fluroquinolones, whilst point mutations in *pbp1* and *16S rRNA* genes have been linked to amoxicillin and tetracycline resistance, respectively [13–15].

While high prevalence of metronidazole-resistant *H. pylori* isolates is common in several places in India like Kolkata, central Gujarat, Chandigarh, Delhi, Lucknow, Hyderabad and Chennai, the rates of *H. pylori* resistance to other antibiotics such as clarithromycin, ciprofloxacin, amoxicillin and tetracycline varied [16–18]. Since the antimicrobial resistance patterns of *H. pylori* vary between different geographical areas, it is vital to implement local antibiotic resistance surveillance program to provide physicians with more informed drug choices to effectively treat *H. pylori*-related gastric diseases. In India, regional antimicrobial resistance profile of *H. pylori* is generally lacking across the nation. Therefore, in this study, we aimed to examine the prevalence of *H. pylori* antibiotic resistance in Karnataka state of India, specifically against five antibiotics including metronidazole, clarithromycin, levofloxacin, tetracycline and amoxicillin. The findings of our work would provide a better understanding of the prevalence of drug-resistant *H. pylori* strains in Karnataka to facilitate the design of more rational and effective antibiotic combinations for eradication of *H. pylori* infection.

## Results

From May 2014 to May 2017, 355 Karnataka patients underwent endoscopy at Kastruba Medical College and Tertiary Care Hospital, among which 180 (50.7%) were positive for *H. pylori* infection based on histopathology examination. A total of 113 *H. pylori* strains were successfully isolated, 80 (70.8%) from male and 33 (29.2%) from female patients. The average age was 46.2 ± 14 years old. Among 113 culture-positive patients recruited from nine districts in Karnataka state, two-third were from Udupi (31.9%, 36/113), Davangere (19.5%, 22/113) and Shimoga (15.9%, 18/113), and the rest were from Bellary (4.4%, 5/113), Chikmagalur (5.3%, 6/113), Chitradurga (10.6%, 12/113), Hassan (0.9%, 1/113), Haveri (4.4%, 5/113) and Uttar Kannada (7.1%, 8/113). Endoscopic examinations further diagnosed 78 (69%), 30 (26.6%) and 5 (4.4%) patients with gastritis, peptic ulcer disease and intestinal metaplasia, respectively. We next examined the susceptibility of each *H. pylori* strain against five different antibiotics including metronidazole, clarithromycin, levofloxacin, tetracycline and amoxicillin. The demographic data of patients and the MIC values for all strains are provided in Additional file 1.

Overall, 14.2% (16/113) strains were susceptible to all antibiotics used in this study (Table 1). Notably, 81.4% (92/113) of isolated strains were resistant to metronidazole. We also observed a high levofloxacin resistance rate among our strains (54.9%, 62/113). Resistance rates towards clarithromycin, tetracycline and amoxicillin were 20.4% (23/113), 5.3% (6/113) and 7.1% (8/113), respectively. Of 67 strains (59.3%, 67/113) that were multidrug-resistant, 58 (86.6%, 58/67) were resistant to at least both metronidazole and levofloxacin. While there was no association between patient ages and the metronidazole resistance rate of isolated clinical strains, metronidazole resistance was moderately lower in females than males (*P *= 0.06) (Table 2). Additionally, resistance to levofloxacin appeared to be the highest in patients aged 60 years and above.

**Table 1:** Antibiotic resistance rates of 113 *H. pylori* strains isolated in this study

Resistance	Total (%)
Overall	
MET	92 (81.4)
CLA	23 (20.4)
LEV	62 (54.9)
TET	6 (5.3)
AMO	8 (7.1)
No resistance	16 (14.2)
Single resistance	30 (26.5)
MET	25 (22.1)
CLA	1 (0.9)
LEV	4 (3.5)
Dual resistance	42 (37.2)
MET + LEV	36 (31.9)
MET + CLA	6 (5.3)
Triple resistance	23 (20.4)
MET + CLA + LEV	13 (11.5)
MET + CLA + AMO	1 (0.9)
MET + LEV + AMO	5 (4.4)
MET + LEV + TET	2 (1.8)
MET + AMO + TET	2 (1.8)
Quadruple resistance	2 (1.8)
MET + CLA + LEVO + TET	2 (1.8)

MET: Metronidazole; CLA: clarithromycin; LEV: levofloxacin; TET: tetracycline; AMO: amoxicillin

**Table 2:** Antimicrobial resistance according to gender and age distributions

Antibiotic resistance	Gender		*P*	Age group			*P*
	Female (n = 33)	Male (n = 80)		20–39 (n = 41)	40–59 (n = 48)	60 and above (n = 24)	
MET	23 (69.7)	69 (86.3)	0.06	32 (78)	42 (87.5)	18 (75)	0.32
CLA	8 (24.2)	15 (18.8)	0.61	5 (12.2)	12 (25)	6 (25)	0.29
LEV	18 (54.5)	44 (55)	1	24 (58.5)	21 (43.8)	17 (70.8)	0.08
AMO	1 (3)	7 (8.8)	0.43	1 (2.4)	5 (10.4)	2 (8.3)	0.33
TET	1 (3)	5 (6.3)	0.67	1 (2.4)	2 (4.2)	3 (12.5)	0.21

MET: Metronidazole; CLA: clarithromycin; LEV: levofloxacin; TET: tetracycline; AMO: amoxicillin

Of 113 strains included in this study, 38 had been sequenced at whole-genome level and reported in our earlier work [19]. Inactivation of RdxA and/or FrxA, nucleotide substitutions in *23S rRNA* gene and amino acid substitutions in GyrA protein, conferring metronidazole, clarithromycin and levofloxacin resistance, respectively, were examined in these 38 strains to determine any of these mutations, if present, matches the susceptibility results. Among 36 sequenced metronidazole-resistant strains, 27 were expected to express non-functional or altered RdxA and/or FrxA proteins resulting from nonsense or frameshift mutations in the coding sequences, and partial gene deletions which would have been likely through recombination (Table 3). In the remaining metronidazole-resistant strains, as well as two metronidazole-susceptible strains, both genes were intact.

**Table 3:** Inactivation mutations in *rdxA* and *frxA* genes in sequenced metronidazole-resistant *H. pylori* strains

Strain	MIC (µg/ml)	*rdxA*	*frxA*
		Mutation type (position)	Mutation type (position)
KH1	64	Nonsense (65)	–
KH2	64	–	–
KH3	64	Nonsense (50)	Nonsense (199)
KH4	16	Nonsense (50)	–
KH6	64	–	Frameshift (70)
KH7	64	–	Nonsense (68)
KH8	64	Frameshift (136)	Frameshift (18)
KH9	32	–	Deletion of 58 bp C-terminal region
KH11	64	–	–
KH12	64	Frameshift (65)	Deletion of 303 bp N-terminal region
KH13	64	–	Frameshift (18)
KH14	64	–	Nonsense (141)
KH15	64	–	–
KH16	64	–	–
KH17	64	Nonsense (50)	–
KH18	64	Fragmented	–
KH19	64	–	Frameshift (44)
KH20	64	–	–
KH21	64	Nonsense (50)	Frameshift (70)
KH22	32	Nonsense (50)	–
KH23	32	–	Frameshift (154)
KH25	64	–	Frameshift (18)
KH26	64	–	Nonsense (68)
KH27	64	–	–
KH28	64	Frameshift (98)	Frameshift (18)
KH29	64	–	–
KH30	64	Frameshift (185)	Nonsense (86)
KH32	16	–	–
KH33	64	–	Frameshift (18)
KH36	32	Frameshift (161)	–
KH37	64	Nonsense (65)	–
KH38	64	Nonsense (50)	Frameshift (18)
KH41	64	–	268 bp insertion
KH43	64	Frameshift (72)	Nonsense (176)
KH44	64	–	–
KH45	64	–	Frameshift (106)

Mutations in the *23 rRNA* gene were detected in all nine clarithromycin-resistant sequenced strains, predominantly A2143G and G2224A single mutations (Table 4). One strain contained double mutations of A2142G and G2224A in its *23 rRNA* gene. Out of 31 levofloxacin-resistant strains, 18 (58.1%) had amino acid substitution occurred at D91 in GyrA, eight (25.8%) at N87 and one at both positions (Table 5). We also noticed that two strains exhibited an unprecedented N-terminal extension of GyrA by five amino acid residues (QDNSV), immediately after the start codon, and one showed a mutation at R295.

**Table 4:** *23S rRNA* gene mutations in nine sequenced clarithromycin-resistant *H. pylori* strains

Strain	MIC (µg/ml)	Mutation(s)
KH6	1	A2143G
KH16	1	A2143G
KH19	1	A2143G
KH20	1	G2224A
KH21	1	G2224A
KH29	1	A2143G
KH35	1	G2224A
KH36	1	A2142G, G2224A
KH41	1	G2224A

**Table 5:** GyrA mutations in sequenced levofloxacin-resistant *H. pylori* strains

Strain	MIC (µg/ml)	Mutation(s)
KH1	4	D91N, N87K
KH2	4	R295H
KH3	4	D91N
KH6	4	D91G
KH8	4	D91N
KH9	4	D91N
KH12	2	D91N
KH13	4	D91N
KH14	4	N87I
KH15	4	D91G
KH16	4	–
KH17	4	D91G
KH18	4	N87K
KH19	4	D91N
KH20	4	5-amino-acid N-terminal extension
KH21	4	D91N
KH25	4	D91Y
KH26	4	N87K
KH27	4	D91Y
KH28	4	D91Y
KH30	4	D91N
KH32	4	D91G
KH33	4	D91N
KH36	4	N87K
KH37	4	D91N
KH38	4	N87K
KH40	4	5-amino-acid N-terminal extension
KH41	4	N87K
KH43	4	D91N
KH44	4	N87K
KH45	4	N87K

## Discussion

To our knowledge, this is the first study to examine the prevalence of antimicrobial resistance of *H. pylori* in Karnataka state of South India. In India, the prevalence of metronidazole-resistant *H. pylori* is remarkably high, with resistance rates reaching at least 80% in several areas [16–18, 20, 21]. Importantly, such high occurrence of metronidazole resistance was also observed in our isolated clinical strains, indicating that conventional triple therapy consisting of metronidazole should not be used to treat *H. pylori* infection in Karnataka. While the main mechanisms conferring *H. pylori* with metronidazole resistance involving RdxA and/or FrxA inactivation mutations were present in 75% of our sequenced metronidazole-resistant strains, in the remaining strains there were considerable number of missense mutations especially in *rdxA* gene that we cannot possibly rule out their role in metronidazole resistance by inducing protein conformational changes and hence dampening the reduction–activation activity of RdxA towards metronidazole [11, 22, 23].

In Maastricht V/Florence consensus report, it was recommended that clarithromycin-based triple therapy should be avoided if local clarithromycin resistance rate exceeds 15% [24]. As the primary clarithromycin resistance rate in Karnataka was 20.4%, which is above the 15% limit, suggesting that clarithromycin-based triple therapy is also not an appropriate option for the treatment of *H. pylori* infection in Karnataka. Resistance to clarithromycin is generally associated with point mutations in the *23S rRNA* gene particularly at positions 2142 and 2143 [25]. In this study, the predominant mutations were A2143G and G2224A, where the latter has been linked to *H. pylori* clarithromycin resistance in the northeast part of China [26].

In tandem with a remarkably high prevalence of metronidazole resistance and a moderate occurrence of clarithromycin resistance, more than half of our isolated *H. pylori* strains were also resistant to levofloxacin, in agreement with prior reports of high levofloxacin resistance rates in several developing nations including Nepal, Vietnam and Bangladesh [27–29]. Levofloxacin resistance in *H. pylori* is acquired primarily by amino acid substitution mutations in GyrA protein involving N87 and D91 [30]. Similarly, 87.1% (27/31) of our sequenced levofloxacin-resistant strains were found to harbour these frequently reported mutations. Of four remaining strains, interestingly, two contained an insertion of QDNSV residues next to the start codon in their GyrA protein. It is highly likely that this five-amino-acid insertion would induce conformational changes in GyrA protein, reducing its binding affinity to fluoroquinolones and thus rendering levofloxacin ineffective. Further, we found one strain that had a R295H mutation in GyrA. Further mutagenesis study would be of interest to confirm the role of these novel GyrA mutations in *H. pylori* resistance to fluoroquinolones including levofloxacin.

Bearing in mind the high to modest rates of *H. pylori* resistance to metronidazole, levofloxacin and clarithromycin in this study, the use of triple therapy containing either combination of these drugs will likely result in treatment failure and should therefore be avoided. Our findings also suggest that triple therapy containing amoxicillin, tetracycline and PPI can be used as one of the first-line regimens for treating *H. pylori* infection in Karnataka. In a recent study conducted in Shanghai, China, where *H. pylori* metronidazole and clarithromycin resistance rates were 74.2% and 24.2%, respectively, at similar levels to Karnataka, the use of quadruple therapy containing bismuth, high-dose metronidazole to overcome existing resistance, clarithromycin and PPI had high modified intention-to-treat (ITT) and per-protocol (PP) cure rates of 90.3% and 96%, respectively [31]. In another study conducted in Zhejiang, China, where *H. pylori* clarithromycin resistance is prevalent, bismuth-based quadruple therapy containing amoxicillin and clarithromycin was shown to achieve both ITT and PP eradication rates of 86.1 and 92.3%, respectively [32]. Hence, it is worth examining the use and the effects of the above-mentioned bismuth-based quadruple therapies for the treatment of *H. pylori* infection in Karnataka population. If proven effective, local medical professionals would be able to continue using both relatively inexpensive metronidazole and clarithromycin drugs as part of *H. pylori* eradication regimens in Karnataka.

## Conclusions

In our study, high resistance rates of metronidazole and levofloxacin resistance, and a modest occurrence of clarithromycin resistance were revealed in *H. pylori* strains isolated from patients in Karnataka, providing important information that triple therapies based on these drugs are not useful as first-line treatment in this South India region. Both amoxicillin and tetracycline are still useful for eradicating *H. pylori* infection in Karnataka. We also revealed novel mutations in GyrA protein that possibly contribute to *H. pylori* resistance in levofloxacin, which merit further investigations.

## Methods

### Gastric biopsies collection

This study was approved by the Manipal University Human Ethics Committee (Ref. No. IEC301/2014). Gastric biopsies were obtained for both histopathological examination and *H. pylori* culturing with informed and written consent from dyspeptic patients who underwent endoscopy at Kastruba Medical College and Tertiary Care Hospital, Manipal (KMC) from May 2014 to May 2017. In total, 113 *H. pylori* clinical strains were successfully isolated from patients who lived in Karnataka state of South India, had not previously received any H_2_ receptor blocker, PPI or nonsteroidal anti-inflammatory drug (NSAID) medication, and without any history of gastric surgery and *H. pylori* eradication treatment, were recruited. Four biopsy specimens (two each from antrum and corpus) were collected in Bouin’s fluid for histopathological examination. Two additional specimens (one each from antrum and corpus) were collected in transport medium for bacterial isolation.

### Bacterial culturing

Transport medium containing biopsy specimens was vortexed prior to plating 100 µl of the solution on brain heart infusion agar (BHIA) supplemented with 5% (vol/vol) horse serum (Gibco, New Zealand), 0.5% (vol/vol) BBL IsoVitaleX enrichment medium (Becton, Dickinson and Company, USA), trimethoprim (5 µg/ml) and vancomycin (6 µg/ml) (HiMedia Ltd., India). The plates were then incubated in a microaerobic atmosphere (5% O_2_, 10% CO_2_ and 85% N_2_) at 37 °C for a period of 3 to 7 days. Successful *H. pylori* isolate was identified based on its distinctive colony morphology, appearance of Gram-negative bacillus under the microscope, positive urease, oxidase and catalase reactions, and PCR detection of *H. pylori*-specific *glmM* gene.

### Minimum inhibitory concentration (MIC) determination using agar dilution method

Plates were prepared containing 5% (vol/vol) horse serum and serial two-fold dilutions of each antibiotic. The range of final concentrations for each antibiotic were 2–64 µg/ml for metronidazole, 0.125–1 µg/ml for clarithromycin, 0.25–1 µg/ml for amoxicillin, and 1–4 µg/ml for both levofloxacin and tetracycline. Bacterial suspension with turbidity of 2.0 McFarland standard was prepared from a 24-h old culture plate in saline. Two µl of bacterial suspension was inoculated onto each antibiotic plate, followed by incubation in a microaerobic atmosphere at 37 °C for 3 days. The MIC was then determined at the lowest antibiotic concentration where bacterial growth was completely inhibited. According to the guidelines of European Committee on Antimicrobial Susceptibility Testing (EUCAST), bacterial isolates are considered to be antibiotic-resistant if the MIC value is > 8 µg/ml for metronidazole, > 0.5 µg/ml for clarithromycin, > 0.12 µg/ml for amoxicillin, and > 1 µg/ml for both tetracycline and levofloxacin.

### Mutation analysis

Thirty-eight draft genomes were annotated using Prokka v1.12 [33]. The Prokka-annotated draft genomes are available at the public data repository Figshare (https://figshare.com/), with 10.6084/m9.figshare.8016128. To obtain *rdxA*, *frxA*, *23S rRNA* and *gyrA* nucleotide sequences from each strain, blastn search of *hp0954*, *hp0642*, *hpr01* and *hp0701*, respectively, from *H. pylori* 26695 reference genome was performed on CLC Genomic Workbench 11 (Qiagen, Germany). Extracted *rdxA*, *frxA* and *gyrA* genes were translated into amino acid sequences prior to alignment. For extracted *23S rRNA* genes, sequence alignment was performed at the nucleotide level. Aligned sequences were then compared between resistant and sensitive strains, with reference sequence included, to examine for reported and novel mutations.

### Statistical analysis

For statistical analysis, Fisher’s exact test and Fisher-Freeman-Halton’s test were used, as appropriate. Only *p* value of < 0.05 was considered statistically significant.

## Additional file

**Additional file 1.** Patient demographics and MIC values. This file contains the minimum inhibitory concentrations of all tested *H. pylori* clinical strains and the demographic characteristics of patients recruited in this study.

## Data Availability

Data are available upon request from the corresponding author (EGC).
